# Oncological and functional impact of adjuvant treatments after open partial laryngeal surgery: a systematic review of the literature and a meta-analysis

**DOI:** 10.1007/s00405-023-07871-8

**Published:** 2023-02-21

**Authors:** Luca Giovanni Locatello, Serena Jiang, Lixiao Chen, Saverio Caini, Giandomenico Maggiore, Pin Dong, Oreste Gallo

**Affiliations:** 1 Department of Otorhinolaryngology, Careggi University Hospital, Florence, Italy; 2 Department of Otorhinolaryngology, University Hospital “Santa Maria Della Misericordia”, Azienda Sanitaria Universitaria Friuli Centrale (ASUFC), Udine, Italy; 3 Department of Otolaryngology-Head & Neck Surgery, Shanghai General Hospital, School of Medicine, Shanghai Jiao Tong University, Shanghai, People’s Republic of China; 4 Cancer Risk Factors and Lifestyle Epidemiology Unit, Institute for Cancer Research, Prevention and Clinical Network (ISPRO), Florence, Italy; 5 Department of Experimental and Clinical Medicine, University of Florence, Florence, Italy

**Keywords:** Adjuvant radiotherapy, OPLS, Laryngeal cancer, Laryngeal surgery, Functional outcomes, Partial laryngectomy, PORT

## Abstract

**Background:**

The oncological and functional role of postoperative radiotherapy (PORT) after open partial laryngeal surgery (OPLS) remains debatable.

**Methods:**

A systematic review and a meta-analysis of the literature were conducted according to the PRISMA guidelines. Outcomes of patients receiving OPLS with and without PORT for laryngeal cancer were summarized.

**Results:**

In the 10 studies that were included in the meta-analysis, no significant difference emerged in terms of pooled overall survival between OPLS patients who did and who did not receive PORT (− 0.3%, 95% CI − 5.4 to 4.9%, *p* = 0.922). Only one study showed a significantly higher incidence of complications in the PORT cohort.

**Conclusions:**

PORT may apparently be performed after OPLS in face of adverse postoperative features without an increased risk of toxicities affecting the neolarynx. Because of the limitations in the available literature, the oncological and functional effects of PORT in this setting needs to be prospectively assessed to strengthen the evidence of this treatment strategy for laryngeal cancer.

## Introduction

Laryngeal cancer (LC) remains a protean disease in terms of biological behavior and its many patterns of diffusion depending on the anatomical location [1]. Traditionally, for early-stage LC (T1-T2 N0) a single-modality strategy is recommended, either with surgery or radiotherapy (RT), thus leaving other treatment options open in case of recurrence [2]; on the other hand, resectable, advanced-stage LC (T3-T4 N +) is usually managed with a multi-modality approach, either a combination of chemotherapy (CHT) and RT (chemoradiation, CRT), or surgical resection followed by adjuvant RT and/or CHT [3].

Open partial laryngeal surgery (OPLS) consists of a large array of surgical procedures that are meant to eradicate LC, while preserving laryngeal functions (breathing without a tracheostomy, speaking, etc.). OPLS has very specific indications and, only in this particular subset of patients, the oncological outcomes may be considered non-inferior to transoral laryngeal surgery or total laryngectomy (TL); this holds true also in the salvage setting, albeit with lower evidence [1, 4]. Unfortunately, OPLS often comes at the cost of persistent voice and swallowing functional impairments that can affect up to 50% of these patients depending on their definition [5, 6]. In this regard, because of preoperative incorrect staging or unexpected pathological features (positive surgical margins, extranodal extension, etc.), a non-negligible proportion of patients receiving OPLS is faced with the need to receive adjuvant RT and/or CHT to improve locoregional control and survival [7–9]. However, the benefits of adjuvant treatments were never specifically investigated in the OPLS population while there is conflicting evidence regarding a higher risk of both short-term (e.g., chondronecrosis) and long-term (e.g., chronic aspiration, neolaryngeal stenosis, etc.) RT-related complications [10–12].

The aim of the present review is to summarize the currently available evidence on the oncological and functional impact of adjuvant RT ± CHT after OPLS while highlighting the unmet needs in this context.

## Materials and methods

### Searching strategy and selection criteria

Following the Preferred Reporting Items for Systematic Reviews and Meta-Analyses (PRISMA) guidelines [13], we conducted a literature search on articles published from January 1980 up to December 2021, using PubMed and Scopus databases to identify the relevant studies. This study was conducted by following the Preferred Reporting Items for Systematic Reviews and Meta-Analyses (PRISMA), and it was registered in the International Prospective Register of Systematic Reviews (PROSPERO, University of York, UK; registration number CRD42021247403).

The following keywords were used: ((adjuvant radiotherapy OR postoperative radiotherapy) AND laryngeal cancer) OR (postoperative radiotherapy AND ( laryngeal tumor OR laryngeal cancer)) OR (partial laryngectomy AND (postoperative radiotherapy OR postoperative radiation therapy)) OR (conservative laryngeal surgery AND postoperative radiotherapy) OR (larynx salvage radiotherapy).

Only studies describing the clinical outcomes of patients with laryngeal squamous cell carcinoma treated with OPLS and, at least in a subgroup, with PORT were included. Articles were excluded based on the following criteria: studies with less than 10 patients or case reports; treatment modalities that involved neoadjuvant CRT, TL, or transoral laryngeal surgery; data not clearly stating the oncological and functional results of the group/subgroup of patients who received adjuvant RT; articles not written in English, French, Italian, or Chinese.

### Data collection

The title and abstract of the selected papers were carefully read according to the inclusion and exclusion criteria and duplicates were removed. Three reviewers (SJ, LXC, and LGL) independently extracted data from each study, which were reviewed for consistency among the authors, and any discrepancies were resolved by consensus. The full text of the included studies was then read to extract the following data:

- Reference: first author, year of publication, and country;
- Recruitment time span;
- Time from surgery to adjuvant RT (weeks);
- Study size and number of patients receiving PORT;
- TNM stage;
- Type of OPLS;
- The mean radiation dose delivered (Gy);
- Complications related to PORT;
- Mean and/or median follow-up time (months);
- Survival outcomes.

### Definition of the outcomes, synthesis of the literature, and meta-analysis

Our initial aim was to compare the oncological outcomes (by all possibly available endpoints such as Overall Survival-OS, Disease-Specific Survival-DSS, and Disease-Free Survival-DFS) between the group receiving only OPLS (i.e., excluding any possible case of salvage procedure) versus OPLS and PORT. After the extraction of all relevant articles, however, we noticed that only a very limited proportion of articles reported these figures separately for the two groups. It was thus decided to critically discuss all the articles qualitatively and to meta-analyze only these latter papers.

Regarding the functional outcomes, we decided to extract all available data about the complications affecting the neolaryngeal function that were attributable only to PORT. We have thus annotated the rates of neolaryngeal dysfunction (i.e., chronic aspiration, aspiration pneumonia, time to and proportion of decannulated patients) in the two groups, to extrapolate the functional parameters after PORT such as the rate of patients that have a sufficient oral intake, time until PEG or nasogastric tube removal, and the proportion of patients requiring them in a permanent manner.

When an open partial horizontal laryngectomy (OPHL) was performed, it was classified according to the nomenclature system introduced in 2014 by the European Laryngological Society (ELS): Type I (supraglottic), Type II (supracricoid), and Type III (supratracheal) with the conservation (a) or sacrifice (b) of the epiglottis and/or the resection of one arytenoid, the base of the tongue, or pyriform sinus (+ ARY, + BOT, + PIR) [14].

### Quality assessment and statistical methods

The quality and the risk of bias of the articles included in the meta-analysis were evaluated by the Quality In Prognosis Studies (QUIPS) tool with any discrepancies resolved by consensus by the first two authors. Visualization of the risk-of-bias assessments was performed by creating a traffic lights plot and a weighted bar plot using the *robvis* tool [15].

The study-specific differences in % OS between patients treated with surgery + PORT vs. patients treated with surgery only (taken as the reference group) were pooled into a summary OS difference by fitting meta-analysis models. A positive % difference meant that the OS was better among patients treated with surgery vs. PORT, and vice versa when the % difference was negative. The between-studies heterogeneity was quantified using the *I*^2^ statistics, which can be interpreted as the proportion of the total variability across studies that is attributable to actual heterogeneity rather than chance. In the case of large heterogeneity (*I*^2^ ≥ 50), random-effect models were fitted, while fixed-effect models were preferred in absence of large heterogeneity. All statistical tests were performed using Stata (command *metan,* Stata Statistical Software: Release 15. College Station, TX, USA: StataCorp LLC) and R (Version 4.1.0, R Foundation for Statistical Computing, Vienna, Austria).

## Results

**Fig. 1:**
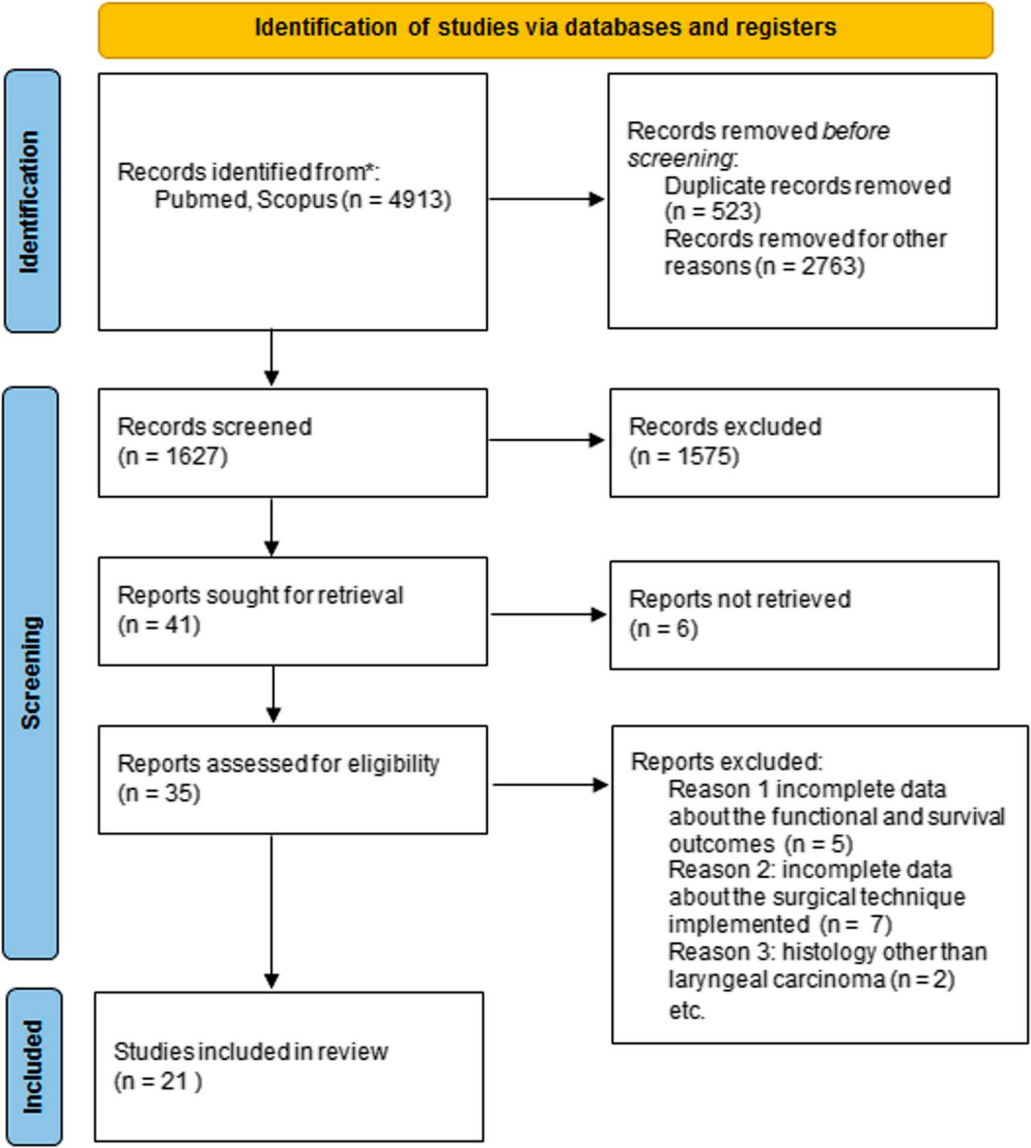
Flow diagram depicting the selection of the papers included in the present review

4913 records for laryngeal cancer and adjuvant PORT were identified from a primary literature search: after the removal of duplicates and by applying the aforementioned criteria, a total of 1627 publications were selected. Papers were then screened by reading the titles and abstracts, and 35 manuscripts were deemed eligible for possible inclusion. After reading the full texts, 14 articles were excluded because of insufficient or incomplete data regarding the surgical and non-surgical treatments received, the survival and functional outcomes, or for histology other than squamous cell carcinoma; only 21 studies eventually met the inclusion criteria. The flowchart presenting our literature search strategy is shown in Fig. 1.

6 studies were carried out in Italy, 6 in China, 1 in Turkey, 1 in Switzerland, 1 in France, 3 in the USA, 1 in Spain, and 2 in Greece. A total of 1959 patients undergoing OPLS were considered, and 970 of them (49.5%) received PORT: a detailed description of their clinical features and oncological outcomes is given in Table 1.

**Table 1:** General description of all the included studies (21)

Reference (year, country)	Treatment period	*N*° of cases (sex)	cTNM/pTNM	Type of open partial laryngeal surgery (%) and proportion of patients undergoing ND	*N*° of patients who received PORT and reason for PORT	Time from surgery to PORT (weeks)	Median radiation dose, in Gy, to the remnant larynx & cervical lymph nodes (range)	Mean follow-up time (range in months)	Survival Outcomes
Costa et al. (2016, Italy) [16]	1984–2012	85 (NA)	cT1-T2N + M0 cT3N0-1M0 /NA	OPHL I ± BOT ± PIR (in T3) ND 100% (87% selective dissection, 13% radical nodal dissection)	85 (all for PSM)	NA	60 (44–70) Nodes with extracapsular extension 56 (46–60) and uninvolved at risk nodes 46 (40–52)	59 (2–280)	1-year DFS 81% 5-year DFS 62% Local recurrence 17%, nodal recurrence 11%, distant metastases 17%, rescue TL 3%
Buglione et al. (2015, Italy) [17]	1984–2012	30 (NA)	cT1-2N(?)M0 /NA	NA ND 63.3%	30 (all for PSM)	NA	60 (44–70)/46 (40–52)	65 (0–220)	Median OS 118 months DSS 190 months DFS 195 months Local recurrence 18%, nodal recurrence 7%, metastases 4%, rescue TL 4%
*Lai et al. (2013, China) [18]	1990–2005	59 (57 M, 2 F)	cT1-4N0-3M0 /NA	OPHL II 15.3% ND 27%	33 (all for PSM)	NA	40–80	44.9 (6.23–129.03)	3-year OS: 62.6% with PORT, 62.6% without PORT 5-year OS: 43.8% with PORT,40.5% without PORT Recurrence rate: 15,1% with PORT, 38,5% without PORT
Guzzo et al. (2010, Italy) [4]	1976–2006	134 (126 M, 14 F)	cT1-4N0-2M0/ NA	OPHL I 66.4% OPHL I + BOT 4.3% OPHL IIa 2.1% OPHL IIb 18.6% Frontolateral laryngectomy 8.6% ND 88% (57 bilateral, 29 unilateral)	39 (53.8% for PSM, 46.2% for nodal disease)	NA	NA	72 (1–295)	5-year DFS 88% for surgery alone, 5-year DFS 83% for OPLS + PORT
*Liu et al. (2009, China) [19]	1990–2003	221 (217 M, 4 F)	cT1-4N0-3M0 /NA	OPHL I 11.76% OPHL II 15.84% ND 67%	121 (for PSM and N +)	NA	40–66	62 (13–194)	5-year DFS:46.4% with PORT, 39.4% without PORT 2-year DFS: 62.5% with PORT, 0% without PORT 2-year OS:90.6% with PORT, 14.3% without PORT
Oksuz et al. (2008, Turkey) [20]	1980–2003	79 (77 M, 2 F)	NA /pT1-4N0-2M0	OPHL I 88.6% OPHL I + BOT 11.4% ND 41.7%	79 (38 for pT3-T4, 25 for PSM, 9 for close margins,19 for unknown margins status, 21 for nodal disease)	6,7 (3–20)	50 (48–70)	NA	5-year locoregional progression-free 86% 5-year DFS 79% 5-year OS 75% 5-year freedom from distant metastasis rate 92% Metachronous second primary tumour 8.8% Died of laryngeal cancer 19% Died of intercurrent disease 12.7%
*Yu et al. (2006, China) [21]	1990–1998	65 (64 M,1 F)	cT3N0-1M0	OPHL II 30% OPHL III 55% ND 5%	25 (for PSM)	NA	NA	> 36	3-year OS: 73,92% without PORT, 80,00% with PORT 5-year OS: 66,33% without PORT, 66.67% with PORT
*Tian et al. (2006, China) [22]	1991–2000	202 (199 M, 3 F)	cT3-4N0-3M0	NA	83 (for PSM)	NA	60–80	60–137	5-year OS: 53.41% without PORT, 51.04% with PORT
Bron et al. (2005, Switzerland) [12]	1981–1999	75 (75 M, 5 F)	cT1-3N0-3M0/pT1-3N0-3M0	OPHL I 100% ND 100%	25 (8 PSM, 14 nodal disease, 3 both)	< 6	45–60	48 (24–199)	5-year DFS: 86.6 without PORT, 94.8% with PORT 5-year local control 90.6 without PORT, 95.8 with PORT
*Esposito et al. (2002, Italy) [23]	1979–1991	97 (95 M, 2 F)	cT1-4N0-3M0 (only 1 N3) / pT1-4N0-3M0	OPHL I 100% ND 100%	35 (NA)	4	64.5 (60–70)	60 (36–180)	5-year OAS 74% (CAS 90%) without PORT; 5-year OAS 61% (CAS 80%) with PORT
*Zeng et al. (2001, China) [24]	1982–1993	87 (85 M, 2 F)	cT1-2N0M0 /NA	OPHL I 4.6% OPHL II 95.4%	20 (16 for PSM, 4 unclear)	NA	50–75	> 60	DFS (positive margin 58.63 ± 10.95, negative margin 146.68 ± 11.03); 5-year OS (positive margin 108.22 ± 16.13, negative margin 156.24 ± 8.05; negative margin); 5-year OS: 90,3% with PORT, 91,2% without PORT
*Zeng et al. (2000, China) [25]	1985–1994	171 (167 M, 4 F)	cT1-4N0-1M0 /NA	OPHL I 14.6% OPHL II 85.4% ND 22.2%	50 (all for PSM)	NA	51–70	> 60	5-year OS: 85.7% without PORT, 79,5% with PORT
Laccourreye et al. (2000, France) [26]	1974–1987	90 (88 M, 2 F)	cT1-3N0-3M0	OPHL I 68.9% OPHL II 31.1% + ARY 27.8% ND 97.8%	90 (4 close margins, 21 advanced T stage, 1 PSM, 24 nodal metastasis, 9 nodal metastasis with extracapsular spread, 57 prophylactic of possible residual micrometastasis in the neck)	6.4 (2.7–12.3)	51.2 (25–71) /50.6 (15–70)	> 120	5-year actuarial survival 71.5% 10-year actuarial survival 41.3% 15-year actuarial survival 36.3% distant metastasis 10% metachronous second primary tumor 43.3%
Spriano et al. (2000, Italy) [11]	1980–1994	56 (55 M, 1 F)	NA /pT1-4N0-2c	OPHL I 59%, OPHL I + BOT 41% ND 91%	56 (9 for PSM, 34 for T3-T4, 28 for > pN2, and 12 for extracapsular spread)	6.8 (2.4–21.6)	50 (40–64)/46 (40–64)	132 (33.6–202.8)	5-year OAS 61 ± 7% 10-year OAS 45 ± 7% 2-year actuarial locoregional control rate 85 ± 5% 5-year actuarial locoregional control rate: 83 ± 5% 2-year ultimate locoregional control rate 87 ± 5% 5-year ultimate locoregional control rate 85 ± 5% Survival rates without any laryngeal impairment: 50 ± 7%, 43 ± 7%, and 39 ± 7% at 2, 5 and 10 years, respectively
*Steiniger et al. (1997, USA) [27]	1980–1990	29 (25 M, 4 F)	cT2-4N0-2M0 /NA	OPHL I 100% ND 88%	17 (for T3-T4, N + , or based on”the discretion of the surgeon.”)	7.9 (3.7–12.4)	57.7 (50–66)	52.3	2-year OS 100 without PORT, 88% with PORT 5-year OS 67% without PORT, 60% with PORT
*Suarez et al. (1995, Spain) [28]	1978–1989	193 (193 M)	cT1-4N0-3M0 /NA	OPHL I 100% ND 98.4%	94 (for N + cases)	< 12	≥ 50	NA	3-year AS 74.5% without PORT, 74.8% with PORT 3-year OAC 74,8% Local recurrences 10.6%, regional relapse 12.8%
Nikolaou et al. (1993, Greece) [29]	1970–1989	66 (64 M, 2 F)	cT2-3N0-2M0 /NA	OPHL I 100% ND 12.1%	24 (PSM, G3 SCC, and pN > 1)	NA	NA	114 (24–228)	29.2% recurrences 20.8% second primary lesion
Daniilidis et al. (1992, Greece) [30]	1976–1989	81 (81 M)	cT2-3N0-1	Vertical OPLS 100% ND 4.9% (N1)	10 (all for PSM)	NA	NA	87.6 (18- 168)	70% locoregional recurrence and metastases
Wang et al. (1990, USA) [31]	1973–1987	24 (16 M, 8 F)	cT2-4N0-2M0 /pT2-4N0-2M0 Stage II–IV	OPHL I 66.7% Vertical OPLS 33.3% ND 62.5%	24 (for PSM and N +)	5.5 (1–9)	59.2 (50.3–71.4)	51.6 (24–144)	5-year actuarial survival rate 62% 5-year recurrence-free survival 67% 5-year overall survival 67% 5-year locoregional control rate 80% Locoregional recurrence 16.6% Distant metastasis 16,6%
*Bocca et al. (1987, Italy) [32]	1970–1980	74 (NA)	cT3-T4/NA	OPHL-I + BOT 30%, + ARI 34%, + PIR 15%, other OPHL-I 21%	13 (all for T4 tumors)	NA	NA	NA	5-year actuarial survival rate for OPLS alone 78.6%, for OPLS + PORT 54%
Burstein et al. (1985, USA) [33]	1969–1982	41 (25 M, 16 F)	cT1-4N0-3M0 /NA	OPHL I ± BOT ND 75%	17 (pN > 1)	NA	50–60	> 24	Locoregional recurrence 11,8%

*PORT* post-operative radiotherapy, *NA* not available, *OPHL* open partial horizontal laryngectomy, *BOT* base of tongue, *PIR* pyriform sinus, *ND* neck dissection, *PSM* positive surgical margins, *DFS* disease-free survival, *TL* total laryngectomy, *OS* overall survival, *DSS* disease specific survival, *CAS* corrected actuarial survival, *OAS* overall actuarial survival

*Denotes the studies included in the meta-analysis

**Fig. 2:**
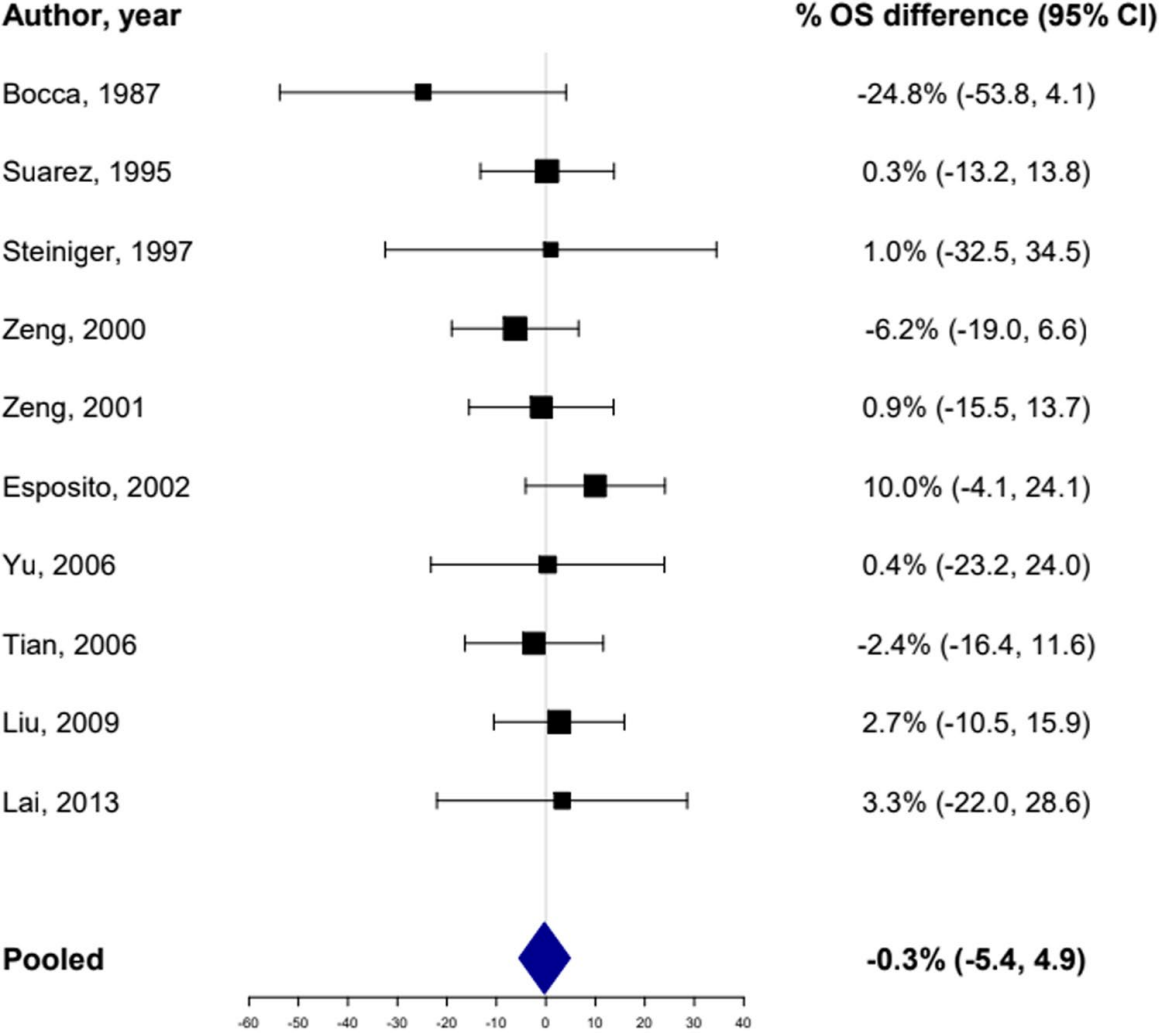
Forest plot representing the pooled OS difference between patients who received PORT after OPLS and those who did not

**Fig. 3:**
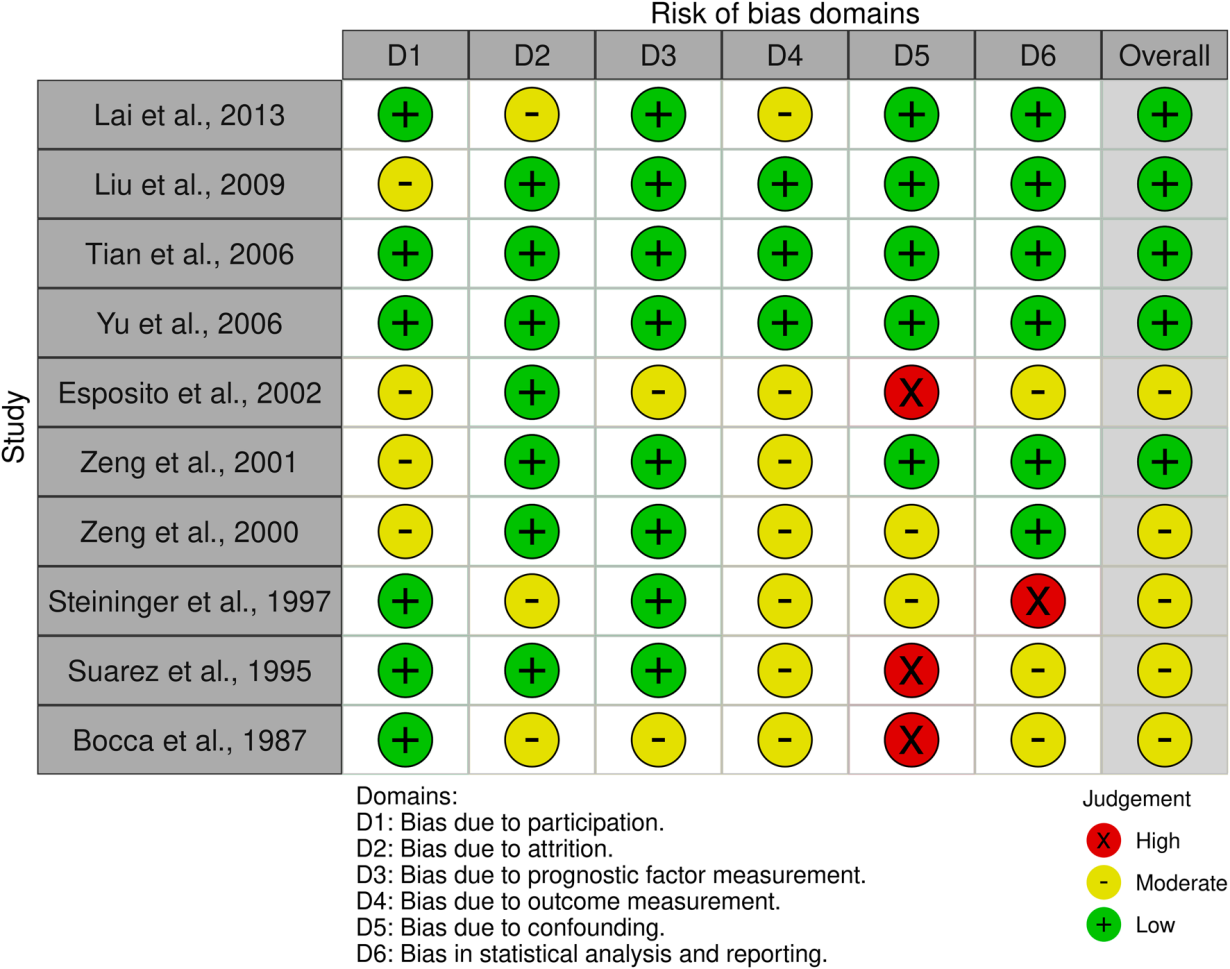
A “traffic light” plot of the domain-level judgments for each result, according to the QUIPS instrument [50]. The plot was generated using the *robvis* tool [15]

**Fig. 4:**
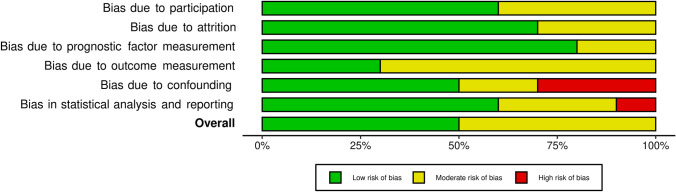
Weighted bar plot of the distribution of QUIPS risk-of-bias judgments within each bias domain [50]. The plot was generated thanks to the *robvis* tool [15]

The treatment period ranges from 1969 to the beginning of 2010 and supraglottic surgery (OPHL I) was the most frequent type of OPLS performed. Indications for PORT on the neolarynx mostly included positive or close surgical margins, and high nodal burden (pN2 or pN3). Only a total of ten articles for a total of 1198 patients reported sufficient information regarding survival separately for those with (491, 40.9%) and without PORT (707, 59.1%). Five-year OS rates greatly differ between these studies, however, no significant difference was found comparing OS for patients receiving or not receiving PORT (pooled %OS = − 0.3%, 95% CI − 5.4 to 4.9%, p = 0.922). No heterogeneity was present (*I*^2^ = 0%) and the resulting forest plot is shown in Fig. 2. Their overall risk of bias was judged to be low to moderate and the traffic lights plot and the weighted bar plot for each domain considered in the QUIPS tool are given in Figs. 3 and 4, respectively.

The collected data about the complications related only to PORT were collected from a total of 14 articles, for a total of 548 patients. They are summarized in Table 2, and exclusively in five articles, a comparison in terms of incidence of complications between the two groups was given. Only in one paper, differences were significant even though outcomes measurements and their definitions largely differed among them.

**Table 2:** Complications related to PORT after OPLS (14)

Reference (year, country)	Sufficient oral intake (%)	Need of NGT/PEG (%)	Time of NGT (months)	Comparison between surgery alone vs. surgery + PORT
Costa et al. (2016, Italy) [16]	92	NA	NA	NA
Buglione et al. (2015, Italy) [17]	100	NA	NA	NA
Oksuz et al. (2008, Turkey) [20]	91.1	1.3	NA	NA
Bron et al. (2005, Switzerland) [12]	79	10.3	< 3	NA
Sessions et al. (2005, USA) [34]	78.2	7.9	NA	Overall 37.8% of complications in surgery + PORT and 38 in surgery alone (*p* = NS)
Laccourreye et al. (2000, France) [26]	94.5	2.2	NA	NA
Spriano et al. (2000, Italy) [11]	81	NA	NA	NA
Steiniger et al. (1997, USA) [27]	65	35	34.8	All surgery-alone patients were able to gain an adequate oral intake between 2 and 30 weeks, and none required permanent feeding gastrostomies Time to decannulation: PORT group 62.5% delayed decannulation (> 3 week), 43.7% delayed decannulation (> 3 months) VS surgery-alone group 33% delayed decannulation (> 3 week), 16.6% (> 3 months) (*p* = 0.32 for 3 weeks and .13 for 3 months) Adequate oral intake (mean): PORT group after 34.8 weeks and surgery-alone group after 7.5 weeks (*p* = 0.20) Delayed development of full oral intake > 3 weeks/ > 3 months: PORT group 56.2%/31.2% VERSUS surgery-alone group 41.6%/25.0% (*p* = 0.18/0.52)
Naudo et al. (1997, France) [35]	91	2.5	NA	Time of decannulation between the irradiated and non irradiated groups
Gregor et al. (1996, South Africa) [36]	100	0	NA	PORT with or without bilateral neck dissection did not show an increase in postoperative morbidity
Wang et al. (1990, USA) [31]	91.7	20.8	NA	NA
Spaulding et al. (1989, USA) [37]	93.9	NA	NA	NA
Robbins et al. (1988, USA) [38]	32	8	NA	OPL versus OPL + PORT: aspiration in 33% VS 44%; weight loss (> 10% of body weight) in 0% VS 12%; NGT 0% VS 8%; pneumonia 0% VS 16%; tracheostomy 0% vs. 8%

Please note that in the second column, patients complaining of mild/grade 1 or 2 dysphagia were included

*NGT* nasogastric tube, *PEG* percutaneous endoscopic gastrostomy, *NA* not available, *PORT* post-operative radiotherapy, *VS* versus, *OPL* open partial laryngectomy

## Discussion

OPLS represents an effective function-preserving treatment for both early and advanced laryngeal cancer but, despite the recent improvements in both endoscopic and imaging techniques, incorrect staging may occur in around 15–20% of patients [40]. It has been demonstrated that PORT with or without concurrent CHT significantly improves survival and reduces the risk of locoregional recurrence when adverse features such as positive margins, high tumoral (pT3-T4), or nodal stage (pN2-N3) are found after surgical resection [8, 9]. However, these results were based on a quite heterogeneous population of head and neck cancer patients [9]. In the context of OPLS, the oncological evidence of the role of PORT remains weak: while some authors showed that PORT can effectively reduce the postoperative loco-regional failure rates to around 8–10% [41], in a retrospective analysis of the US National Cancer Database on 1460 surgically treated LC patients (where only 90 underwent OPLS and 23 OPLS + PORT), in pT3N0R0 LC cases no survival benefit was found with adjuvant treatments (adjusted HR, 0.88; 95% CI 0.64–1.21) [42]. In the present work, we found no difference in terms of OS for patients receiving or not receiving RT, but we must recall that patients treated with PORT likely show a more advanced disease (*i.e.*, they are considered to have a worse prognosis) compared to those treated with surgery alone. Therefore, since in the meta-analyzed studies it was not possible to obtain separate data on patients' characteristics or other relevant prognostic factors, our result might be indirectly interpreted in favor of PORT as a positive prognostic factor for OPLS patients.

There are some conflicting issues, which are specific to this combined “surgical preserving” plus RT strategy [43–48]. When OPLS is applied in improperly selected patients, achieving a R0 resection may become very difficult without converting to TL, and this explains the frequent encounter of close/positive surgical margins. [44]

Intriguingly, an American study where PORT was deliberately avoided in favor of a wait-and-see approach for R1 cases revealed that, despite a higher local recurrence rate in the former compared to the R0 cases, survival outcomes were notably the same in the two groups [45]. Then PORT is often requested after OPLS because of the postoperative finding of microscopic extracapsular extension in the excised lymph nodes which is a well-established adverse prognostic factor [46] and whose incorporation into the latest AJCC/UICC TNM edition has inevitably led to rising in the pathological stage [48]. On the contrary, it is known that adjuvant RT should ideally be started within 6 weeks, [9] but OPLS patients are typically characterized by the need for a long postoperative functional rehabilitation (for OPHL II, the mean reported length of hospital stay ranges from 5 to 104 days) [43]. Besides the need for proper recovery of the neolarynx, another reason not to irradiate these patients is given by the worsening of persistent laryngeal edema that was shown to increase the clinical difficulties in detecting recurrence in a large cohort of OPLS + PORT [20], even though the prognostic significance of this aspect remains understudied.

In the present paper, and in accordance with other authors [10, 34], we think that concerns about the potentially deleterious effects of PORT in OPLS might have been overemphasized. While it is true that in some series a worsening of neolaryngeal function was demonstrated after RT (longer tracheostomy dependence [4, 27], the longer time to resume oral intake/more frequent need to place a PEG [38, 41, 48], etc.), the exact contribution of PORT to these adverse events remains hardly discernible. It is well established that functional recovery depends on several factors, including age and the type of OPLS with vertical/hemilaryngectomy showing better swallowing outcomes compared to OPHL, while the removal of one cricoarytenoid unit significantly increases the postoperative hospital stay [43, 48]. On the other hand, some complications could be undoubtedly attributed to PORT such as cricopharyngeal stenosis [12], laryngeal stenosis [26, 39], or chondronecrosis [11]. It must be remembered, however, that these events can occur even in non-irradiated OPLS patients: for example, laryngeal stenosis can be attributed to arytenoid edema, posterior prolapse of the epiglottis, mucosal webs, and cicatricial narrowing of the pexy [49–52].

If a major strength of our study is the comprehensive evaluation of the literature, without limiting it to the Western surgical series, limitations in the included articles have substantially hindered our research because of several reasons. Underreporting of data was a common finding given that most of the works are retrospective analyses of OPLS series where a variable proportion of patients received PORT. An unavoidable obstacle was also represented by the many technical variations that go under the OPLS definition: though these operations can be ultimately tailored to the single case, the impact of neolaryngeal reconstruction on the incidence of complications after PORT could not be ascertained. Another critical issue was the heterogeneity of the included population with different treatment protocols, anatomical subsites involved, and follow-up protocols. Finally, in the recent decades, the evolution of RT techniques must be accounted for as the latest dose-delivery protocols have substantially changed to spare in a more precise manner the non-involved tissues: this indeed represents a limit in the analysis of PORT toxicities [50–52]. In summary, though a direct perspective comparison does not yet exist in terms of survival, an indirect benefit for PORT may be derived from our results. However, the many unreported factors in the available literature (poor performance status of patients making them unfit for PORT, the surgical quality declined as number of OPLS procedures performed annually, etc.) strongly limit a sound retrospective comparison for these two groups of patients.

## Conclusions

In the treatment of laryngeal squamous cell carcinoma, from the present meta-analysis, it appears that PORT can be effectively performed after OPLS in face of adverse postoperative features, and without an increased risk of toxicities affecting the neolarynx. Unfortunately, the level of evidence regarding the oncological role of PORT in this setting remains low because of the limitations in the available literature and this holds also true for the functional complications of RT, whose techniques have greatly evolved in the latest years. Only specifically designed clinical trials investigating the role of PORT after OPLS will ultimately define the strengths and the drawbacks of this combined strategy in the management of intermediate- and advanced-stage LC.

## Data Availability

Data of the present research are available upon reasonable request to the Corresponding Author.
